# Pediatric Myeloid Sarcoma of the Testes Treated With Surgery and Adjuvant Radiation Therapy

**DOI:** 10.7759/cureus.57029

**Published:** 2024-03-27

**Authors:** Steven Miller, Julian Jeberaeel, Anas Saad, Nour Abd almohsen, Nitin Vaishampayan

**Affiliations:** 1 Department of Oncology, Wayne State University School of Medicine, Detroit, USA; 2 Department of Pathology, Wayne State University School of Medicine, Detroit, USA

**Keywords:** 3d conformal radiation therapy, urologic oncology, radiarion oncology, pediatric solid tumours, acute myeloid leukemia (aml), testicular myeloid sarcoma

## Abstract

Myeloid sarcoma (MS) is a rare extramedullary tumor of immature granulocytic cells and is most often associated with acute myeloid leukemia (AML). Myeloid sarcomas can occur anywhere in the body but are seldom present in the testicles, especially in the pediatric population. The treatment of MS, especially testicular myeloid sarcoma (TMS) is not well defined in the literature and the role of radiation therapy in the treatment of TMS is even less well defined. In this case report, we discuss the treatment for TMS in a pediatric patient, review the literature, and discuss the role of radiation therapy in the treatment.

## Introduction

Myeloid sarcoma (MS) is an extramedullary neoplasm composed of immature cells derived from a myeloid hematopoietic lineage. These neoplasms can have many names, and some of the most common in the literature, include granulocytic sarcoma, monocytic sarcoma, extramedullary myeloid cell tumor, myelosarcoma, myeloblastoma, and chloroma [1]. Testicular myeloid sarcoma (TMS) is a very rare entity with available literature limited to case reports and a few review articles with a small number of patients. The incidence of TMS in patients with MS is only about 4%- 6.5% as reported in a few institutional case series [1-3]. Secondary to the limited data for this disease, the treatment recommendations for MS, especially TMS are relatively limited. Indications, guidelines, and recommendations for radiation therapy in the treatment of TMS are even more limited.

For this case study, we report on a nine-year-old male who underwent a hematopoietic stem cell transplantation (HSCT) for acute myeloid leukemia (AML). He subsequently developed a TMS involving the left testicle two years after transplant for which he underwent a left orchiectomy and adjuvant radiation therapy. He is over nine months out since the completion of radiation therapy with no evidence of recurrent disease.

## Case presentation

The patient is a nine-year-old male who was diagnosed with high-risk AML. He initially presented with weight loss, fever, bruising, and shortness of breath. He was found to be anemic with a hemoglobin of less than 4 g/dl at presentation and flow cytometry and a bone marrow biopsy were consistent with AML. He was initially treated with decitabine, idarubicin, fludarabine, cytarabine, and granulocyte colony-stimulating factor (G-CSF). He underwent an HSCT approximately five months after diagnosis and received a preparative regimen of busulfan and cytoxan followed by stem cell infusion. He received post-transplant cytoxan and did well with treatment. 

**Figure 1:**
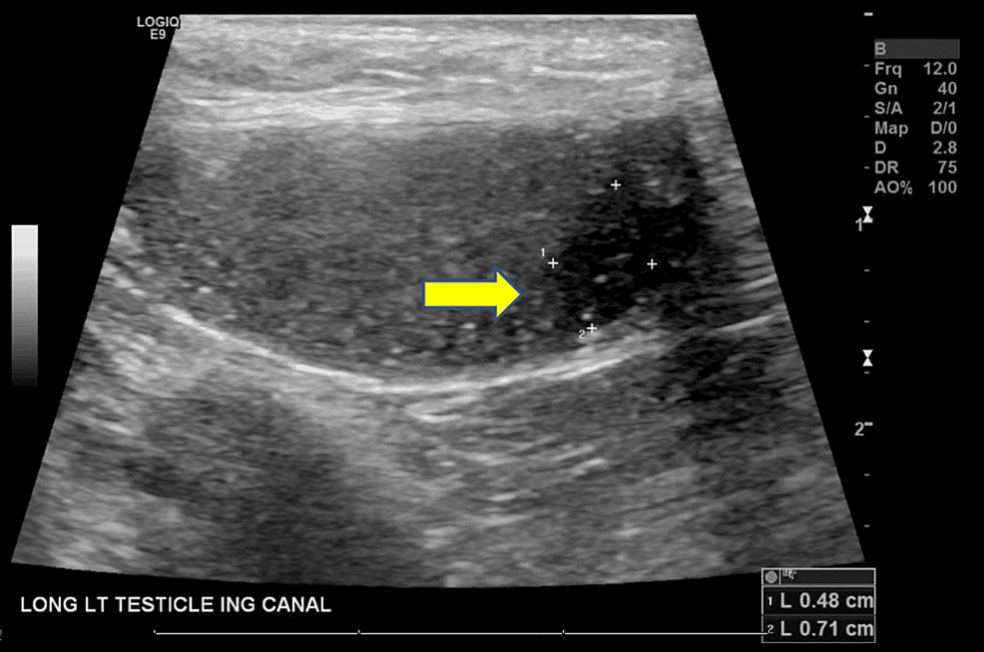
Ultrasound image of the left testicle The arrow is pointing toward the 0.5 x 0.7 x 0.5 cm hypoechoic lesion in the inferior pole.

**Figure 2:**
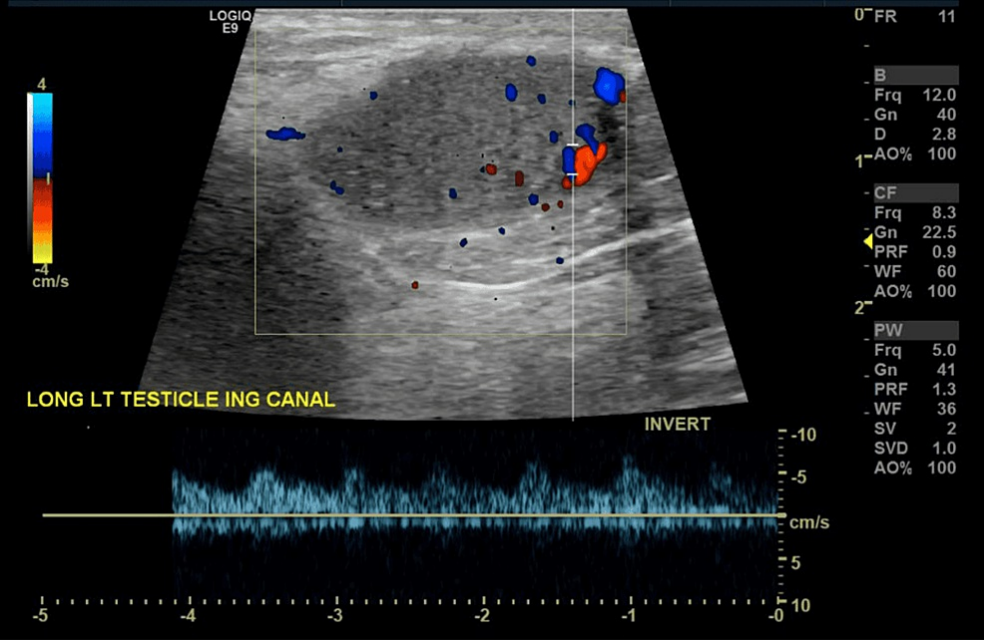
Color Doppler study image Scattered punctate hyperechoic foci and central color Doppler vascularity can be seen involving the inferior pole of the left testicle.

Approximately two years after the transplant, he developed swelling and enlargement of his left testicle with no associated pain or discoloration. He also denied any recent trauma to the left testicle. The left testicular swelling persisted, and he subsequently underwent a scrotal ultrasound at the request of his oncologist. The scrotal ultrasound revealed an enlarged left testicle measuring 2.4 x 1.2 x 1.1 cm with a volume of 2.2 mL. In the inferior pole of the left testicle, a 0.5 x 0.7 x 0.5 cm hypoechoic lesion was noted. Scattered punctate hyperechoic foci and central color Doppler vascularity were also seen in the inferior pole of the left testicle which was concerning for a malignancy (Figures 1, 2). No appreciable hydrocele fluid or scrotal fluid collection was noted. 

The right testicle measured 1.5 x 0.8 x 1.0 cm with a volume of 0.85 mL. There was no evidence of mass or fluid collection within the right testicle or scrotum and no appreciable hydrocele fluid. He underwent a left radical orchiectomy soon after the ultrasound, and a pathologic review of the left testicle revealed a dense infiltration of malignant cells as well as blasts with high nuclear-to-cytoplasmic ratios. Finely dispersed chromatin, prominent nucleoli and high mitotic activity were also noted. 

**Figure 3:**
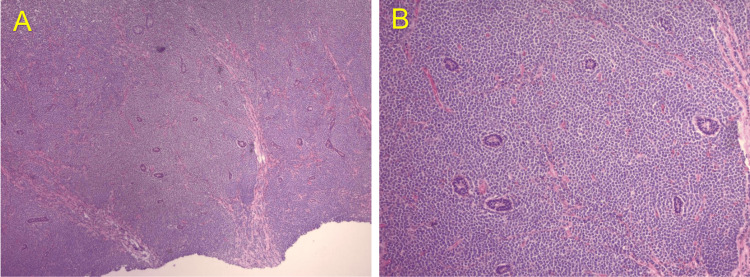
Immunohistochemical study images (I) (A) H&E 4X. Histology showing dense infiltration of malignant cells. (B) H&E 10X. Higher power showing blasts with high nuclear-to-cytoplasmic ratios, finely dispersed chromatin, and prominent nucleoli.

**Figure 4:**
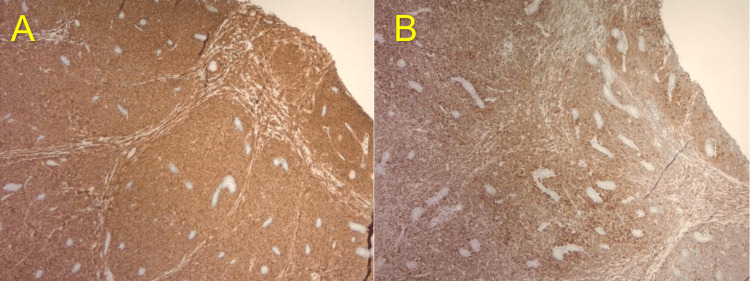
Immunohistochemical study images (II) (A) CD43 immunostain positive for myeloid blasts and (B) Myeloperoxidase (MPO) immunostain positive for myeloid blasts.

A panel of immunohistochemical stains was performed. The neoplastic cells were positive for CD43 (strong, diffuse), myeloperoxidase (MPO) (intermediate to strong), CD117 (moderate, diffuse), CD99 (moderate, diffuse), CD79a (weak to moderate, patchy), and CD3 (weak to moderate, patchy) which was consistent with MS (Figures 3, 4). Stains were also negative for cytokeratins, ruling out carcinoma, negative for synaptophysin, ruling out neuroendocrine origins, and negative for SALL4 and OCT 4, ruling out germ cell origin.

A positron emission tomography (PET) scan obtained two weeks after surgery revealed no evidence of any FDG avid malignancy including the left orchidectomy site. A lumbar puncture was negative, and a bone marrow biopsy was unremarkable.

**Figure 5:**
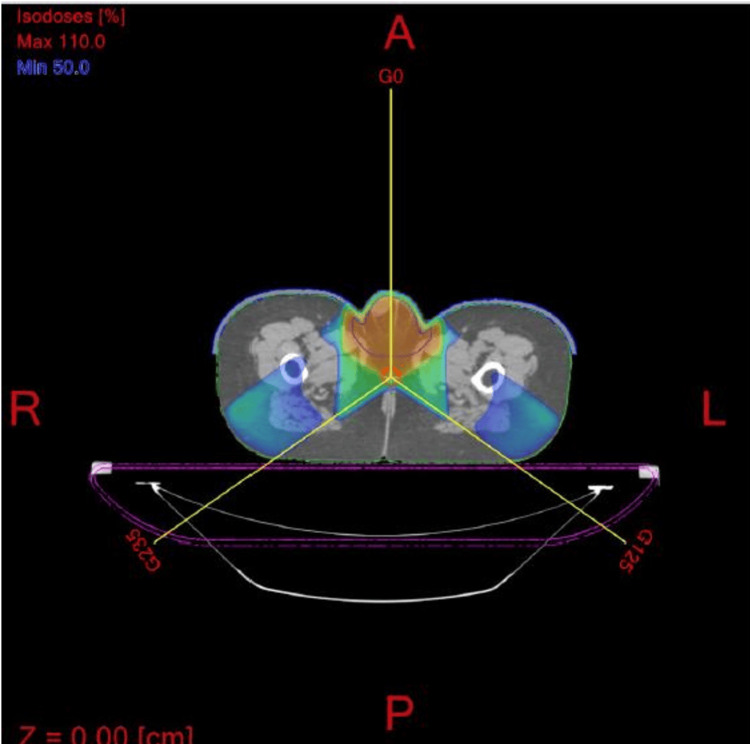
Radiation therapy fields Radiation therapy fields treating the scrotum (three-field technique consisting of an anterior field and two oblique fields). The 100% radiation dose region is in red and the radiation beams have been designed to avoid the rectum and the bladder.

**Figure 6:**
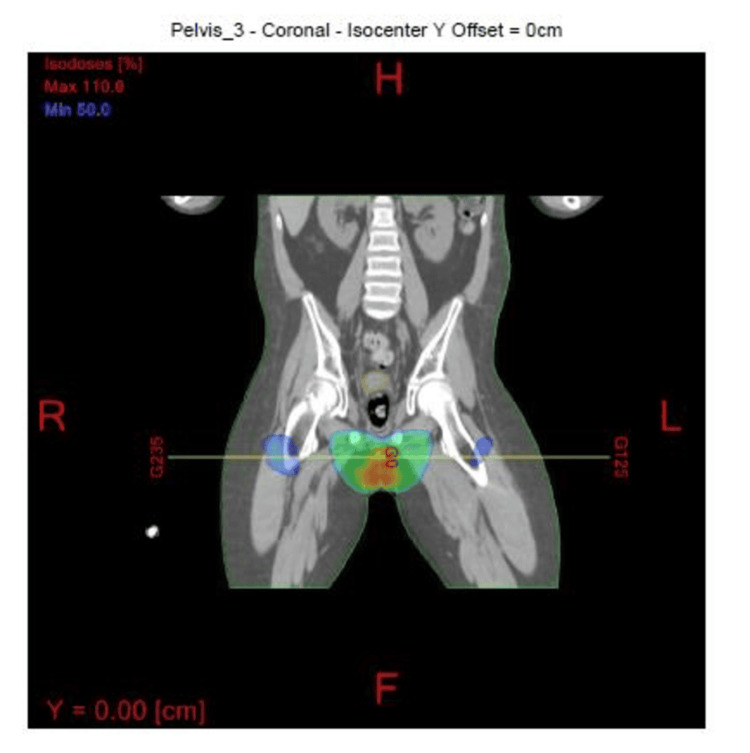
Coronal view of the radiation fields Radiation fields with 100% radiation dose volume in red and 50% in green.

The patient underwent a course of adjuvant radiation to the left testicular bed and scrotum. The radiation fields were designed to treat the scrotum and right testicle. A three-field radiation technique was designed to cover the entire scrotum including the right testicle. Since the left testicle was removed, a Gross Tumor Volume (GTV) was not defined. The Clinical Tumor Volume (CTV) was the scrotal sac and the right testicle. The Planning Target Volume (PTV) was a 1 cm margin around the CTV. The radiation fields consisted of an anterior field and two oblique fields with energies of 6 and 10 MV photons (Figures 5, 6). The entire scrotum and the right testicle were treated secondary to concerns of recurrent disease developing in the left scrotal sac as well as concerns that the right testicle may be a sanctuary site for recurrent disease.

**Figure 7:**
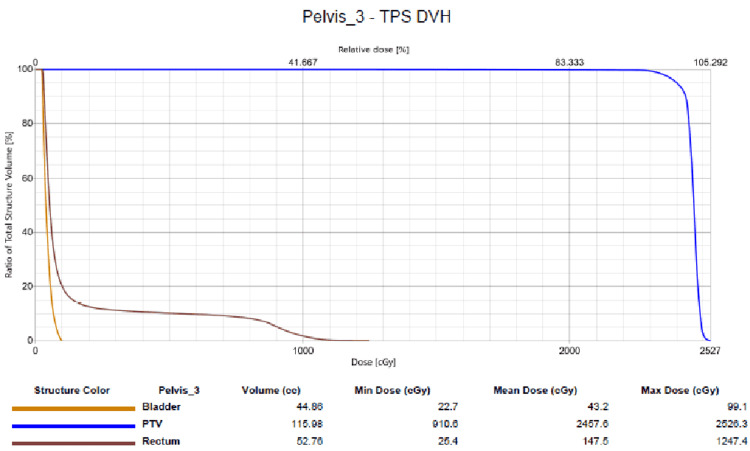
Dose volume histogram Dose volume histogram (DVH) is displaying the doses administered to the critical structures, including the bladder, rectum, and planning target volume (PTV).

The patient received a total dose of 2400 centigray (cGy) in 12 fractions to the PTV with a 0.5 cm bolus placed over the scrotum to help increase the skin surface dose. A cradle was fashioned to stabilize the pelvis and the penis was secured superiorly and placed out of the radiation field. The doses to the rectum and bladder were within the dose constraints for these organs. The mean dose to the bladder, rectum, and PTV was 43 cGy, 147 cGy, and 2457 cGy. The maximum dose (Dmax) to the bladder, rectum, and PTV was 100 cGy, 1155 cGy, and 2533 cGy (Figure 7). The overall dose constraints were the following: 3000 cGy to less than 80% of the bladder and 3500 cGy to less than 50% of the bladder. No dose constraint was applied to the right testicle since it was included in the radiation treatment volume.

The patient tolerated radiation therapy well overall, with no significant skin reactions, side effects, or treatment breaks. A PET scan obtained approximately two months after radiation therapy revealed no evidence of metastatic disease or disease recurrence in the scrotum. The patient is now over nine months status post-radiation therapy with no evidence of recurrent disease noted on recent imaging.

## Discussion

Myeloid sarcomas can present at multiple sites in the body including the skin, bone, central nervous system (CNS), and testis, with skin being the most common site [3]. Myeloid Sarcomas in general, usually occurs as a secondary manifestation, either before or simultaneously with AML. It is also encountered rarely as MS or a primary chloroma and may precede AML by months or years. Myeloid sarcoma can also be an independent entity, without progression to a hematologic disease [4]. The testes are often considered a sanctuary site for AML and acute lymphoblastic leukemia (ALL). The existence of a physiologic blood testis barrier that prevents the entry of large-molecular weight compounds into the seminiferous tubule is thought to explain this, but this is still a subject not clearly understood [5].

Myeloid Sarcoma overall has a greater incidence in the pediatric population than in the adult population, with an incidence of approximately 30% in children with AML compared to a lower percentage of 2% to 5% in the adult population [6]. Extramedullary relapses of AML after HSCT are rare and uncommon. Excluding the CNS, when extramedullary relapses do occur, they have been described to occur in locations such as the skin, gums, and soft tissue [7]. Isolated extramedullary relapses are described in a very small number of cases, occurring in about 0.65% of patients who were allo-grafted for AML and 3.7% of patients allo-grafted for AML or chronic myeloid leukemia. Bone marrow relapses do occur and can develop 1-12 months after an extramedullary relapse and, in rare cases, can be cured by local treatment [7].

Imaging of testicular lesions with ultrasound and or a PET scan can be valuable in the diagnosis of a testicular lesion. Myeloid sarcomas have been found to have a moderate FDG uptake of 2.6-9.7 [8]. Ultrasound is commonly used to evaluate testicular swelling or a testicular mass and has been shown to have a greater than 90% sensitivity and specificity [9]. An MRI can also serve as a useful imaging modality when ultrasound findings are uncertain or when the location of an intrascrotal mass is unclear. A CT scan with contrast is typically used for nodal staging, especially in evaluating the retroperitoneal lymph nodes.

The differential diagnoses for pediatric testicular swelling should include lymphoma, multiple myeloma, testicular Leydig cell tumor, rhabdomyosarcoma, Sertoli cell tumors, gonadoblastoma, epidermoid cysts, and inflammatory pseudotumor [8]. An extensive antibody panel should be performed for the diagnosis of MS [1]. Staining for myeloperoxidase (MPO) has been used in the past to help differentiate MS from lymphoma. Myeloperoxidase is expressed in 66% to 96% of MS and is also responsible for its green appearance on exposure to the air, leading to its historic name “chloroma” [1].

Histologically, MS presents as a dense infiltrate of malignant cells in the extramedullary tissue, which can be mistaken for other types of non-hematopoietic neoplasms based on morphology alone. Higher magnification often reveals a population of blasts with varying degrees of myeloid differentiation. These cells typically have high nuclear-to-cytoplasmic ratios, with the nuclei being round to irregular in shape, and displaying finely dispersed chromatin and prominent nucleoli. The diagnosis of myeloid sarcoma is confirmed through immunohistochemical staining for myeloid lineage markers such as MPO, CD43, and CD117. 

One of the largest series concerning TMS is by Sahu et al., in which they reviewed all published literature concerning TMS. In their series, they were able to evaluate 68 cases of TMS. Of the 68 cases, no data was available in 18 cases [10]. In the 50 cases that remained, 22 (44%) had left-sided and 18 patients (36%) had right-sided TMS involvement. The remaining 10 patients (20%) had bilateral involvement, with six patients having simultaneous bilateral involvement, while the remaining four patients had sequential involvement of one testicle after the other. The most common presenting symptom for these patients was scrotal swelling or a scrotal mass. Malignancies that were most commonly associated with TMS included acute myeloid leukemia (AML), chronic myeloid leukemia (CML), and chronic myelomonocytic leukemia [10]. Most patients in this series were treated with various treatment modalities including combinations of chemotherapy, surgery, radiation therapy, and HSCT. Radiation doses of 350-2600 cGy were used to treat the testicles as the sole modality or in combination with chemotherapy with the aim of achieving remission and preventing disease recurrence in the contralateral testis [10].

A case report by El Jamal et al. examined a pediatric case of TMS and reviewed the literature. Five pediatric cases of TMS were evaluated with a median age at presentation of 49 months (range, 7.5 to 132 mo.) and a testicular mass was the most common symptom [4]. Eight additional cases in the literature were reported on that were either stand-alone case reports or cases that were embedded in larger case series. A detailed description of the morphologic and immunophenotypical features of pediatric testicular MS was reviewed, especially in the five cases, with all cases demonstrating monoblastic morphology and immunophenotypical features as evidenced by the positivity of CD33, CD43, CD4, CD68, CD163, and lysozyme [4]. Four of the five cases showed KMT2A (MLL) gene rearrangement, which is consistent with the immunophenotype, and morphology observed.

An additional case series by Du et al. was reported on six patients (all adults) who were diagnosed with TMS after homologous stem cell transplantation [1]. Fifteen to 36 months was the time period between transplant and the diagnosis of TMS and four of the six patients had bilateral testicular disease. Two patients underwent a bilateral radical orchiectomy, and the remaining four patients underwent a unilateral radical orchiectomy with three patients receiving radiation therapy to the contralateral testicle. All of the patients received chemotherapy as part of their treatment, and the authors stated that radiation therapy in combination with chemotherapy could be used for consolidation after induction chemotherapy and that his treatment could prevent the recurrence of disease to the contralateral testis, which would be especially important for patients that desire to preserve testicular function. At the time of the last follow-up from the report (13-84 months after diagnosis of TMS), four of six patients were alive [1].

Concerning radiation therapy, a case report by Dahbi et al. describes a technique to deliver radiation to the patient using an immobilization device and a computed tomography scan to help design the radiation fields. A conformal radiation therapy technique was designed to allow for targeted radiation therapy delivery to the testis and scrotum while avoiding other tissues such as the bladder and rectum. This technique helped to ensure that the radiation therapy was localized to the intended target while minimizing radiation exposure to the organs at risk, such as the rectum and bladder potentially decreasing side effects [11].

One of the many challenges in treating a patient with scrotal irradiation is reproducing the treatment setup and ensuring that the patient is in the correct position on a daily basis. The scrotum tends to be a very mobile sac which can be difficult to stabilize and can also change in size due to many factors such as temperature and emotions. To accommodate these variables, the patient should be positioned supine with the legs slightly separated to expose the scrotum. The penis should be placed on the abdominal wall and taped into position. The scrotum is supported and immobilized using a personalized immobilization device with a bolus under and around the scrotum [11]. Concerning radiation dose recommendations for TMS, a paper by Bakst et al. reviewed 38 patients treated at their institution with radiation for MS. The recommended dose for MS from the paper was 2400 cGy in 12 fractions [12]. As far as radiation techniques are concerned, an article by Brouwer et al. evaluated different radiation therapy techniques to treat the scrotum and testicles. Photon irradiation with two oblique beams using wedges resulted in the best coverage per the paper compared to an en-face electron field or opposing radiation fields [13].

Radiation therapy is commonly used in the treatment of MS, but no overall survival benefit has been demonstrated with its use. Tsimberidou et al. reported no benefit in event-free survival with radiation therapy alone [14]. Radiation therapy has been used alone or in combination with chemotherapy to treat MS with the goal of achieving durable remission and hopefully preventing a recurrence in the contralateral testis [9]. Hematopoietic stem cell transplantation is considered the definitive treatment strategy in the management of MS. In retrospective studies, an overall survival benefit has only been observed in MS patients who underwent HSCT. Radiation is generally used in consolidation after induction chemotherapy, for treating localized recurrences after HSCT, and in palliative settings to reduce large tumor masses to relieve compression symptoms and reduce pain [15-16].

## Conclusions

Testicular myeloid sarcoma is a rare entity with very few guidelines available in the literature to describe the most efficient method to treat this disease. Besides surgery and systemic chemotherapy, as well as HSCT, regional radiation therapy to the involved site of the disease can also help with long-term local control. Unfortunately, no overall survival advantage has been demonstrated with the use of radiation therapy in TMS. When treating TMS with radiation, conformal radiation therapy techniques should be used to ensure that an adequate radiation dose is delivered to the site of disease. This radiation technique can also reduce the dose of radiation to the organs at risk such as the bladder and the rectum. This in turn can decrease the side effects of radiation therapy and make the radiation treatments more tolerable for the patients. More data and follow-up information will need to be collected on patients with TMS, but localized radiation therapy to assist in local control should be considered especially in the pediatric population.

## References

[1] Du Y, Li Q, Zhang X, Xu T (2022). Clinical characteristics and treatment outcomes of testicular myeloid sarcomas after hematopoietic stem cell transplantation: a single-institution experience. Clin Genitourin Cancer.

[2] Almond LM, Charalampakis M, Ford SJ, Gourevitch D, Desai A (2017). Myeloid sarcoma: presentation, diagnosis, and treatment. Clin Lymphoma Myeloma Leuk.

[3] Fonseca A, Scheinemann K, Jansen J, Barr R (2014). Testicular myeloid sarcoma: an unusual presentation of infant acute myeloid leukemia. J Pediatr Hematol Oncol.

[4] El Jamal SM, Salama A, Marcellino BK (2020). Myeloid sarcoma of the testis in children: clinicopathologic and immunohistochemical characteristics with KMT2A (MLL) gene rearrangement correlation. Appl Immunohistochem Mol Morphol.

[5] Ginsberg JP, Orudjev E, Bunin N, Felix CA, Lange BJ (2002). Isolated extramedullary relapse in acute myeloid leukemia: A retrospective analysis. Med Pediatr Oncol.

[6] Klco JM, Welch JS, Nguyen TT (2011). State of the art in myeloid sarcoma. Int J Lab Hematol.

[7] Yildirim I, Uçkan D, Cetin M, Tuncer M, Tezcan I (2007). Isolated testicular and bone relapse in children with acute myeloblastic leukemia and chronic graft versus host disease after allogeneic BMT. Turk J Pediatr.

[8] Wu J, Jiang G, Wu J, Ou L, Zhang C (2021). 18F-FDG PET/CT imaging of testicular myeloid sarcoma in a pediatric patient. Clin Nucl Med.

[9] Thomas KL, Jeong D, Montilla-Soler J, Feuerlein S (2020). The role of diagnostic imaging in the primary testicular cancer: initial staging, response assessment and surveillance. Transl Androl Urol.

[10] Sahu KK, Sherif AA, Mishra AK, Lal A, Singh A (2019). Testicular myeloid sarcoma: a systematic review of the literature. Clin Lymphoma Myeloma Leuk.

[11] Dahbi Z Sr, Elmejjabar R, Alami R, Kouhen F (2023). Testicular radiotherapy: a challenging irradiation site. Cureus.

[12] Bakst R, Wolden S, Yahalom J (2012). Radiation therapy for chloroma (granulocytic sarcoma). Int J Radiat Oncol Biol Phys.

[13] Brouwer CL, Wiesendanger EM, van der Hulst PC, van Imhoff GW, Langendijk JA, Beijert M (2013). Scrotal irradiation in primary testicular lymphoma: review of the literature and in silico planning comparative study. Int J Radiat Oncol Biol Phys.

[14] Tsitouridis I, Maskalidis Ch, Pervana S, Pazarli E, Kariki EP (2014). Radiologic and pathologic features of a primary chloroma of the testis: a case report and brief review of the literature. Hippokratia.

[15] Nguyen HT, Terao MA, Green DM, Pui CH, Inaba H (2021). Testicular involvement of acute lymphoblastic leukemia in children and adolescents: diagnosis, biology, and management. Cancer.

[16] Ravikumar D, Ambalavana K, Elumalai SK, Vijayaraghavan N, Ramesh NL (2023). Acute myeloid leukemia masquerading as testicular mass: a case report. Cureus.

